# Complete mitochondrial genome of a parasitic wasp *Microplitis pallidipes* (Hymenoptera: Braconidae: Microgastrinae)

**DOI:** 10.1080/23802359.2020.1715893

**Published:** 2020-01-27

**Authors:** Baoqian Lyu, Rui Meng, Bo Cai, Hao Su

**Affiliations:** a Environment and Plant Protection Institute, Ministry of Agriculture and Rural Affairs, China Academy of Tropical Agriculture Sciences/Key Laboratory of Integrated Pest Management on Tropical Crops, Haikou, China;; b Post-Entry Quarantine Station for Tropical Plant, Haikou Customs District P. R. China/Hainan Province Engineering Research Center for Quarantine, Prevention and Control of Exotic Pests, Haikou, China

**Keywords:** Microplitini, mitogenome, gene rearrangement, phylogenetic relationship

## Abstract

The complete mitochondrial genome of *Microplitis pallidipes* contains 15931 bp, with an A + T content of 86.5% and consists of 13 protein-coding genes, 22 transfer RNA genes, two ribosomal RNA genes, and a control region (GenBank accession no. MN892396). All of the 22 tRNA genes displayed an usual clover-leaf structure. Gene rearrangement events occurred in this species, there are eight tRNA genes changed their positions or/and directions. 13 PCGs started with ATN. Ten PCGs used the typical stop codon ‘TAA’ and ‘TAG’, three PCGs terminated with incomplete stop codons (TA). Phylogenetic analyses within the microgastroid complex were performed based on mitochondrial protein-coding genes.

*Microplitis pallidipes* Szépligeti (Hymenoptera: Braconidae: Microgastrinae: Microplitini) is an important larval parasitoid of noctuid larval pests (You et al. 2006; Gao et al. 2007). It is a solitary endoparasitoid of 1st to 4th instar larvae of the noctuids but prefers the 2nd to 4th instar (Zeng et al. 2005). To date, no mitogenome had been studied for the tribe. 110.336033,19.989214

In this study, the complete mitochondrial genome of *M. pallidipes* is sequenced. Adult samples were obtained in the insectarium of Environment and Plant Protection Institute, China Academy of Tropical Agriculture Sciences, Hainan, China (110°20′9″N, 19°59′21″E). The specimens were deposited at −20 °C in the herbarium of Post-Entry Quarantine Station for Tropical Plant, Haikou Customs District P. R. China (Specimen accesion number IN07070101-0000–0020). A single individual was used for DNA extraction.

The complete circular mitogenome of *M. pallidipes* has a length of 15931 bp (Genbank accession number MN892396). The nucleotide composition of *M. pallidipes* mitogenome was biased toward AT 86.5%, and the total base composition was 42.0% A, 44.5% T, 7.6% G, 6.0% C. The circular genome contained 13 protein-coding genes (PCGs), 22 tRNA genes, 2 rRNA genes, and 1 A + T rich region.

Gene rearrangement events occurred in this species. All rearranged genes were tRNA, not as the *C. vestalis* of Microgastrinae which has seven of 13 protein-coding genes changed their positions or/and directions (Wei et al. 2010). This species has the following tRNA arrangement patterns: trnI(-)–trnM(-)–A + T-rich region–trnQ, trnC- trnY–trnW, trnH(-)– trnD–trnK, trnN– trnA–trnR–trnS1–trnE–trnF and trnV(-)–l-rRNA–s-rRNA.

The length of 13 PCGs was 11,133 bp, all PCGs started with ATN. Ten PCGs used the typical stop codon ‘TAA’ and ‘TAG’, three PCGs terminated with incomplete stop codons (TA). All of the 22 tRNAs have the usual clover-leaf secondary structure. All tRNAs had normal lengths, which varied from 64 to 71 bp. The 16S rRNA was 1140 bp long with an AT content of 89.9%, while the 12S rRNA was 755 bp long with an AT content of 89.5%. The non-coding region (putative control region) was 979 bp in length with an A + T content of 91.5%.

The concatenated datasets of 13 PCGs in seven species representing four subfamilies of microgastroid subfamily complexes were used in phylogenetic analyses (Figure 1). Two species respectively from Sigalphinae and Pselaphaninae were used as outgroups. Phylogenetic analyses were performed with Bayesian inference MrBayes 3.2.3 (Ronquist et al. 2012) and maximum likelihood in RAxML 8.2.10 (Stamatakis 2014). The results of phylogenetic analysis within microgastroid subfamily complexes were similar to the previous study (Li et al. 2016). The result further confirmed that *M. pallidipes* and *Cotesia vestalis* was close to each other, as they are the only two mitochondrial genomes of Microgastrinae reported so far.

**Figure 1.**
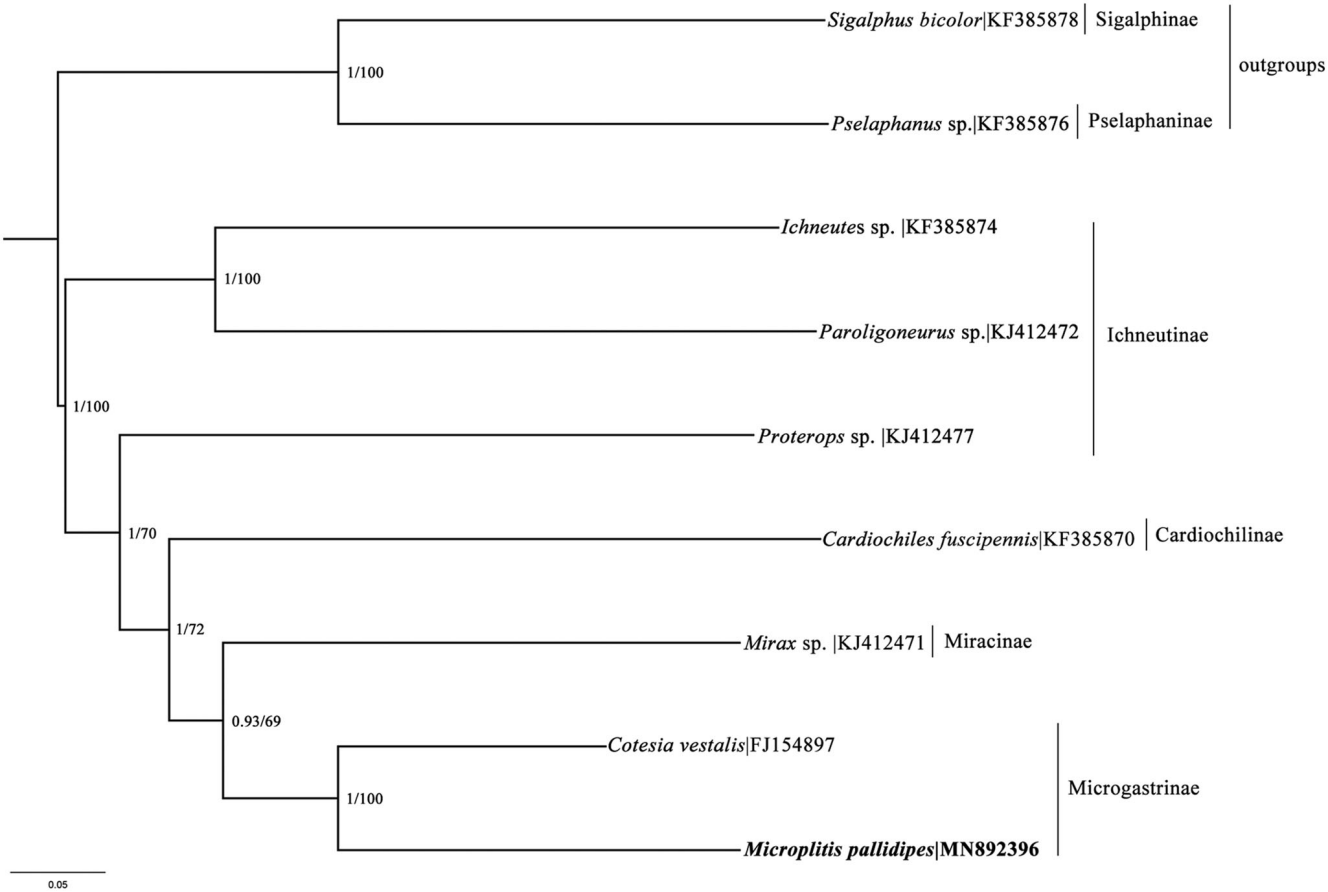
Phylogenetic relationships within the microgastroid complex inferred from nucleotide sequences of mitochondrial protein-coding genes, using Bayesian/ML methods.
